# Searching *Mycoplasma pneumonia* by serology & PCR in children with adenoid hypertrophy and rhinosinusitis: A case control study, Tehran, Iran

**Published:** 2013-03

**Authors:** Samileh Noorbakhsh, Mohammad Farhadi, Azardokht Tabatabaei, Sahar Ghavidel Darestani, Shima Javad Nia

**Affiliations:** 1 Research Center of Pediatric Iinfectious Diseases; 2 ENT –Head & Neck Research Center, Tehran University of Medical Sciences

**Keywords:** Rhino sinusitis, Adenoid tissue, Adenoid hypertrophy, *M. pneumoniae*

## Abstract

**Background and objectives:**

Chronic infection in childhood is a leading cause of adeno-tonsillectomy****. The aim of this study was to determine the role of *M. pneumoniae* in children with rhino sinusitis and adenoid hypertrophy.

**Method and Material:**

This case - control study was carried out in the pediatric and ENT wards of Hazrat Rasul Hospital, Tehran, Iran (2007-2009). In this trial, we investigated 40 cases with adenoid surgery and 32 controls.We looked for *M. pneumoniae* -DNA (PCR) in adenoid tissues resected from cases and 31 nasopharyngeal swabs in controls and IgM & IgG antibodies (ELISA) were compared between the 2 groups, P < 0.05 was considered to show a significant value.

**Results:**

Positive PCR results were observed in 35% of cases and none of controls, positive-IgG was seen in 20% of cases and 6.4% of controls (P = 0.71) which was higher in older cases (6 vs. 4 years, p < 0.05). Positive –IgM was seen in 10% of cases vs. 9.7% of controls, (P= 0.74); without any difference for age (6.2/ 5.3 years, p = 0.1). A positive PCR result was not related to positive IgG (p = 0.014), but to a positive IgM (p= 0.1).

**Conclusion:**

*M. pneumoniae* infection was found serologically (IgM & IgG) in10% and 20% of cases, respectively. These numbers along with positive PCR in adenoid tissue of cases (30%) indicates the prominent role for *M. pneumoniae* in adenoid hypertrophy. We concluded that children in Iran will have been infected with *M. pneumoniae* and would have obtained immunity between the ages of 6 and 8. Adenoid tissue might act as a reservoir for *M. pneumoniae* and cause rhino sinusitis concomitant with adenoid hypertrophy in infected children. Theoretically, suitable *M. pneumoniae* eradicating antibiotics before adenoid surgery (with rhino sinusitis or chronic ear infection) might be helpful treatment, but it needs future RCT studies to be proven.

## INTRODUCTION

Tonsillar tissue is a component of mucosal-associated lymphoid tissue (MALT), which protects mucosal surfaces. Chronic infection in childhood is a leading cause of adeno-tonsillectomy. Chronic rhino sinusitis and adenoid hypertrophy in children might have one etiologic factor. Adeno tonsillectomy is efficient in reducing the number and severity of subsequent episodes of throat infection for at least 2 years (1, 2). The adenoid, which has a central role in the development of secretory otitis media, may act as a reservoir for bacteria, causing ear infection and cholesteatoma (3). Brook *et al*. highlighted the importance of the bacterial load in the adenoids in contributing to the etiology of recurrent otitis media, recurrent adenotonsillitis, and obstructive adenoid hypertrophy (4). Up to 5 to 13% of children may experience sinusitis, but a precise data is not available (4). Early and effective antibiotic treatment is necessary for reduction of infectious period and mucosal injuries and complications. Piacentini *et al*. reported an atypical bacteria in adenoids and tonsils of children requiring adenotonsillectomy (5).

Some studies demonstrated the presence of *M. pneumoniae*in adenotonsillar tissue of children with community acquired infections. *Mycoplasma* spieces are naturally found in adeno tonsillitis (6–8). A number of reports defined the role of other atypical infections; like *M. pneumoniae* in children with rhino sinusitis and adenoid hypertrophy (6–9).

Rhino sinusitis is one of the most common causes of pediatrician visit in our hospital. (10). Previous studies in Tehran proved sinusitis is common in children (11, 12)

PCR is a more sensitive method for detection of *M. pneumoniae* compared with serology (13, 14). Little is known about the role of *M. pneumoniae* in children with rhino sinusitis and adenoid hypertrophy. The aim of this study was to determine its role in children with adenoid hypertrophy accompanied by rhino sinusitis.

## METHODS AND MATERIALS

This case - control study was carried out in the pediatric and ENT wards of Rasoul Akram Hospital in Tehran (2007-2009). It was approved by the Ethical Committee of the ENT Department of Hazrat Rasul Hospital in Tehran University of Medical Sciences. Consent Letter was obtained from patients and controls. Initially a questionnaire was completed by an authorized physician, followed by complete clinical exams.

Our study group consisted of 40 children with rhino sinusitis and adenoid hypertrophy, and 31 controls. All case and controls were younger than 14 years old. Diagnostic parameters for rhino sinusitis were based on clinical and imaging diagnostic parameters for rhino sinusitis criteria (2). The control group consisted of 31 children who were hospitalized for elective general surgery in the general surgery ward (i.e. appendicitis, hernia, etc.). The controls were age matched with cases. They were visited by a pediatrician before surgery to be assessed on rhino sinusitis. Only if they had no manifestation of the disease after appropriate physical exams, they were considered as controls. We used their extra blood (which was taken for their routine blood tests before their respective surgery) for the serologic tests.

### Exclusion criteria

We excluded all cases with immunodeficiency states and those who had received any type of antibiotics at least 2 weeks before surgery. All cases with known malignancy or other causes except infection for adenoid hypertrophy (proved in pathology) were excluded.

Blood samples (2 ml) were obtained from 40 cases and 31 controls and centrifuged. It was transferred and kept frozen at -20°C in our research laboratory. ELISA assay (Biochem Immuno Systems, Italy) for specific IgM and IgG antibodies against *M. pneumoniae* was done. Results were interpreted by cut-off control as suggested by the manufacturer.

Nasopharyngeal swabs were used to detect *M. pneumoniae*-DNA in controls (Ethics restricted in controls). During surgery 1cm of resected adenoid tissue was removed and put down in a sterile tube by the surgeon. All samples were then transferred to the research laboratory right away. They were kept frozen at -80°C until the DNA was extracted (PCR template Purification Kit Roche; Germany). PCR- ELISA kits (Roche, Germany) were used for detecting the *M. pneumoniae*-DNA according to the manufacturer order in Roche Diagnostics. Using this kit, as low as 10 copies/ml of bacterial genome can be detected. We used ELISA reader at 450 nm.

### Statistical analysis

The Student's t test was used to determine significant differences in means for all continuous variables. Chi-square values (CI 95%; P < 0.05) were calculated for all categorical Variables and P < 0.05 was considered to have significant value. All analyses were conducted using SPSS, version 11.5.

## RESULTS

We studied on 40 cases in need of adenoid surgery. They were between 3-14 years old, with a mean age of 8 ± 2 year; (48%) of cases were male; (52%) female. Site of sinus involvement in cases: (10.4%) pan sinusitis; (77.6%) maxillary; (9%) frontal and (3%) ethmoide sinuses.23% of cases went under adenoid surgery in spring, (17%) in summer, (28%) in autumn, and 32% in winter.

*M. pneumoniae*–DNA was detected in 35% (14/40) of cases but not in any of the controls (0/31). Although the mean age of cases with positive *M. pneumoniae* –DNA in adenoid tissue was slightly higher than that of the cases with negative results (8.2 years vs. 7.4 years = 0.6) but there was no significant difference between the two groups. Positive *M. pneumoniae*–DNA was related neither to sex nor to the season of adenoid surgery in cases. Positive PCR results had no significant difference (20% vs. 30% p = 0.6) between cases younger (1/4) and older than 5 years old (12/40).

Positive serum *M. pneumoniae* – IgG did not show a significant difference between cases and controls [20% (8/40) vs. 6.4% (3/31), P= 0.74]. Positive *M. pneumoniae* -IgM was detected in 10% (4/40) of cases compared to 9.7% (3/31) of controls without any significant difference (P= 0.74). Positive and negative *M. pneumoniae* -IgM & IgG results were not related to age, gender in cases. Mean age of cases with positive *M. pneumoniae*-IgG was higher than those with negative results [6 vs. 4 years; p = 0.05] but it was not the case for *M. pneumoniae* - IgM [6 .2 vs. 5.3 years; p = 0.1].

Table-1 compares the serology and PCR results in cases. Positive PCR was higher in cases (p = 0.05) but it was not for serologic resuts (p = 0.7; 0.74).

**Table 1:** Comparison of the test results between cases and controls.

Variable results Test	Case / control	Mean age Positive / negative
*M. pneumoniae*-DNA ( PCR)	35% / 0% p = 0.05	8.2 /3.4 year p = 0.6
*M. pneumoniae* –IgM	10% / 9.7% p = 0.74	6.2/ 5.3 years p = 0.1
*M. pneumoniae* -IgG	20% / 6.4% p = 0.71	6 /4 year ;p = 0.05

P value <0.05 considered statistically significant

Table 2 shows that positive PCR was related to positive IgM results (p= 0.1) in cases but it was not for positive IgG (p = 0.014).

**Table 2:** Comparison of the serology and PCR in cases.

*M. pneumoniae* serology		PCR		Total
	
		Positive	negative	Positive
IGG	Positive	6	2	8
	Negative	8	24	32
Total p value = 0.014		14	26	40
IGM	Positive	6	4	10
	Negative	8	22	30
Total p value = 0.1		14	26	40

P value <0.05 considered statistically significant.

## DISCUSSION

In the present study, the *M. pneumoniae* –DNA was found in resected adenoid tissue of the cases but not in nasopharyngeal tissues of controls (35% vs. 0%, p = 0.05). In spite of greater serologically positive results for recent infection in the cases, the difference was not meaningful (positive IgM 10%, vs. 9.7%; p = 0.7). Indeed recent infection (positive IgM) was observed in older cases with adenoid surgery. Positive IgM result was correlated to positive PCR results (p= 0.1). Good correlation between PCR and IgM test indicates the prominent role of recent *M. pneumoniae* infection in our cases.

In contrast to IgM, positive IgG was not related to positive PCR in cases (p = 0.014), and was also observed in older cases [6 vs. 4 years; p < 0.05]

Due to higher sensitivity of PCR for detecting *M. pneumoniae* compared to serology tests, detection of DNA (35% vs. 0) in adenoid tissues would exaggerate and magnify these little serological differences. Hence, both tests confirm the role of recent *M. pneumoniae* infection in cases with adenoid hypertrophy. Besides, serological studies could differentiate the real infection (positive DNA in adenoid tissue) from contamination of adenoid tissue during surgery with oral cavity flora. However, cases with serologically (6.2 years) or PCR (8 years) proven infections were older than those without infection.

Likewise, Nilsson et al. concluded that for the diagnosis of acute *M. pneumoniae* infection, PCR is superior to serology and reveals higher rates of persistent infection (15). Piacentini *et al*., (5) studied the atypical bacteria in adenoids and tonsils of children requiring adenotonsillectomy. *M. pneumoniae* were isolated from 6 patients (10.9%), which is lower than the present study. The results of study conducted by Huminer et al is close to the present one. Higher rate of infection was observed in children with recurrent adenotonsillitis (34.5%) compared to cases with obstructive symptoms (3.7%).

Freymuth *et al*, (6) explained the role of viral, *C. pneumoniae* and *M. pneumoniae* infections in exacerbation of asthma in children. *M. pneumoniae* – DNA (PCR) was obtained from 3.7% of cases which showed a minor role in exacerbation of asthma (6). In contrast to our study, Sprinkle *et al*, (7) showed *C. pneumoniae* and *M. pneumoniae* are not involved in recurrent or chronic adenonsillitis (unlike acute tonsillopharyngitis), but again, most infections happen in higher age (8.2 years vs. 4.2 years) (7).

In contrast to the developed countries where *M. pneumoniae* infections are generally considered to reach a peak between the ages of 10-12 years, they can occur commonly at an early age (4-6 years old) in our population. Recent infection (positive IgM) was found in 10% of cases. In immune cases (positive *M. pneumoniae*-IgG), it may represent as persistent and chronic *M. pneumoniae* infection in adenoid tissues. *M. pneumoniae* is less commonly detected in other upper respiratory conditions such as otitis media, sinusitis, and the common cold.

This study proved that *M. pneumoniae* can act as one etiologic factor for rhino sinusitis and adenoid hypertrophy. The previous studies in our center confirmed these results (12, 14). *M. pneumoniae* may presents as an asymptomatic upper or lower respiratory infection in children (12). These infections are sometimes asymptomatic. This pattern may have a wide variation in different populations. *M. pneumoniae* may be colonized in adenoid tissue of children or nasal polyps of adults (16). A variety of methods exist for laboratory diagnosis of *M. pneumoniae* infection, including culture, serology, and the polymerase chain reaction assay, but each has its limitations (15). Erythromycin, tetracycline or other new macrolides (azithromycin, clarithromycin) are recommended antibiotics for *M. pneumoniae* infection. We prefer to use a suitable antibiotic treatment in children with rhino sinusitis before resorting to adenoid surgery.

In conclusion, *M. pneumoniae* infection was proven serologically (IgM & IgG) to exist in 10% and 20% of cases in need of adenoid surgery, respectively. These amounts along with the positive PCR results in adenoid tissue of cases (30%) indicate the prominent role of *M. pneumoniae* in adenoid hypertrophy. We concluded that children in Iran are infected with *M. pneumoniae* between the ages of 6-8 years and obtaine immunity by then. Adenoid tissue might act as a reservoir for *M. pneumoniae* and cause rhino sinusitis concomitant with adenoid hypertrophy in infected children. Theoretically, the use of suitable antibiotics to eradicate the *M. pneumoniae*, before performing adenoid surgery (with rhino sinusitis or chronic ear infection) might be helpful but it needs further randomized controlled trials studies to be confirmed.
